# Gender differences of cognitive function in migraine patients: evidence from event-related potentials using the oddball paradigm

**DOI:** 10.1186/1129-2377-15-6

**Published:** 2014-01-27

**Authors:** Rongfei Wang, Zhao Dong, Xiaoyan Chen, Mingjie Zhang, Fan Yang, Xiaolan Zhang, Weiquan Jia, Shengyuan Yu

**Affiliations:** 1 Department of Neurology, Chinese PLA General Hospital, Fuxing Road 28, Haidian District, Beijing 100853, China

**Keywords:** Migraine, ERPs, P3, N2, Gender difference

## Abstract

**Background:**

Migraine shows gender-specific incidence and has a higher prevalence in females. Gender plays an important role in the prevalence of migraine, but few studies have investigated the effect of gender on the cognitive functions of migraine patients. This study investigated gender differences in the cognitive function of migraine patients without aura.

**Methods:**

We recruited 29 migraine patients (15 females; mean age 25.4 y) during the interictal period and 28 healthy age-matched participants (14 females; mean age 24.8 y). We used an auditory oddball paradigm to analyze target processing using event-related potentials.

**Results:**

We investigated the N2 and P3 components. The P3 amplitude was decreased in patients compared with the control, and this reduction was not modulated by gender. These results of the P3 provided a new evidence for the dysfunction of cognitive function in migraine patients. The N2 amplitude was larger for male than female migraine patients, and this gender effect was not found in the control group.

**Conclusions:**

These results of the P3 provided a new evidence for the dysfunction of cognitive function in migraine patients. And those of N2 may explain that male patients have the super-sensitivity of cerebral function relevant to the early target-selection and response preparation. Our findings emphasize the importance of considering gender when researching the cognitive function of migraine patients.

## Background

Migraine is one of the most common types in primary headache. It is characterized by episodic acute and severe disruptions of the brain parenchyma and be accompanied photophobia, phonophobia and gastrointestinal disturbance. As a common disabling primary headache disorder, the migraine has been ranked as the third most prevalent disorder and seventh-highest specific cause of disability worldwide [1]. Interestingly, migraine shows gender-specific incidence and has a higher prevalence in females. The prevalence of migraine is 9.3%, and the female: male is 2.09:1 in China [2].

Previous studies about gender differences in migraineurs found that the influences of migraines on the structures and functions of brain are different for males and females [3,4]. For example, Maleki et al. found that female migraineurs had thicker posterior insula and precuneus cortices than male migraineurs [3]. To date, evidence has revealed that migraine patients showed impairment in cognitive functions such as processing speed, sustained attention [5-7], working memory [8,9], and visual-spatial processing [4,10-12]. Although gender plays an important role in the prevalence of migraine, but few studies have investigated the effect of gender on the cognitive functions of migraine patients, which will be explored in the present study by recording and analyzing the event-related potentials (ERPs).

ERPs reveal coherent stimulus-related postsynaptic activity in the cortex with millisecond temporal resolution and, hence, are ideally suited for investigating the time course of cortical activation for cognitive processing. Several ERP studies have systematically assessed the cognitive function using the P3 component in migraine patients and observed reduced P3 amplitudes [13,14]. In contrast, there was also evidence that, when compared with normal participants, the P3 amplitude was enlarged and delayed in primary headache patients [15]. In addition to the P3, Boćkowski and colleague found longer N2 latencies in migraine patients without aura in comparison with migraine patients with aura and tension-type headaches patients [16]. Importantly, recent ERP studies demonstrated gender effects on N2 and P3 components [17,18]. Because the gender influences the prevalence of migraine, it is necessary to investigate the gender differences on attentional function using ERP components.

## Methods

### Participants

We recruited 29 patients with migraine without aura (15 females; mean age 25.4 y, range between 20 to 30 y) from the Chinese PLA General Hospital according to the International Headache Society (ICHD) criteria. Patients were verified to receive no prophylactic therapy and had to have been drug-free for at least 72 h. Migraine attack frequency was 1–6/month. The time interval between the last attack of migraine and the recording was at least 1 week. We also recruited 28 healthy age-matched participants (14 females; mean age 24.8 y, range between 21 to 30 y) with no history of headache attacks or drug/alcohol abuse. All of the participants had normal or corrected-to-normal vision, and normal hearing capability. No participants had remarkable dysfunctions in their motor and sensory systems, or deep tendon reflexes. We excluded participants who were illiterate, or suffering from depression, stroke, or brain injuries. This study was approved by the Ethical Committee of the Chinese PLA General Hospital, in accordance with the ethical principles of the Declaration of Helsinki. All participants gave their written and informed consents prior to the experiment.

The following clinical data were included for the migraine patients: 1) the history of the migraine, 2) the frequency of headaches per month over the previous year, 3) a rating of the most severe headache experienced in the previous year using a visual analog scale (VAS), and 4) with photophobia and phonophobia during the migraine attacks. The exclusion criteria were: 1) taking prophylactic medications for migraine, 2) a history of analgesic drug overuse, 3) general neurological or psychiatric disease, 4) a history of drug abuse or dependency, including that related to alcohol consumption and cigarette smoking, 5) a history of mixed headache types, 6) a history of a neurological disorder or abnormal findings on a neurological examination. There were no significant gender differences in the durations of the migraine history (t(1,29) = 0.08, p = 0.94), the migraine frequencies (t(1,29) = 0.30, p = 0.77), and the VAS scores ( t(1,29) = 0.06, p = 0.96).

### Stimuli and procedures

The experiment was performed in a sound attenuated room with dim light. The stimuli included 1600-Hz (target, 20% probability) and 1,000-Hz (standard, 80% probability) pure tones, with linear rise and fall times of 5 ms and with an intensity of 65 dB. Both stimuli were presented through headphones unilaterally, with the duration of 105 ms. The interstimulus interval (ISI) varied randomly from 1000 to 1500 ms (mean, 1200 ms). There were two separate blocks of 160 stimuli for each.

Participant was instructed to focus on a fixation cross in the center of the screen and to press the button as quickly and correctly as possible when they heard the target stimuli.

### EEG recording and analysis

Electroencephalogram (EEG) was continuously recorded (band pass 0.05-100Hz, sampling rate 500Hz) at Fz, Cz and Pz electrode sites according to the international 10–20 system with ASA-Lab EEG/ERPs 64 Chanel Amplifier (http://www.ant-neuro.com), referenced to the left mastoid (right mastoid as recording site). VEOG and HEOG were recorded with two pairs of electrodes, one placed above and below the right eye, and the other 10 mm from the lateral canthi. Electrode impedance was maintained below 5 kΩ throughout the experiment.

We used ASA software (http://www.ant-neuro.com) to analyze the data off-line. EEG data were re-referenced to the bi-mastoid average reference. EOG artifacts were corrected using the method proposed by Semlitsch et al. (1986). The EEG was segmented into the epoch from 200 ms pre-stimulus to 1000 ms post-stimulus. The EEG segment contaminated by amplifier clipping, bursts of electromyographic activity, or peak-to-peak deflection exceeding ±100 μV were excluded from averaging. The EEG segments were averaged separately for target and standard stimuli. The number of average trials left after removal of the artifacts was 60 (target) and 210 (standard) for normal controls and 64 (target) and 206 (standard) for patients, respectively.

**Figure 1:**
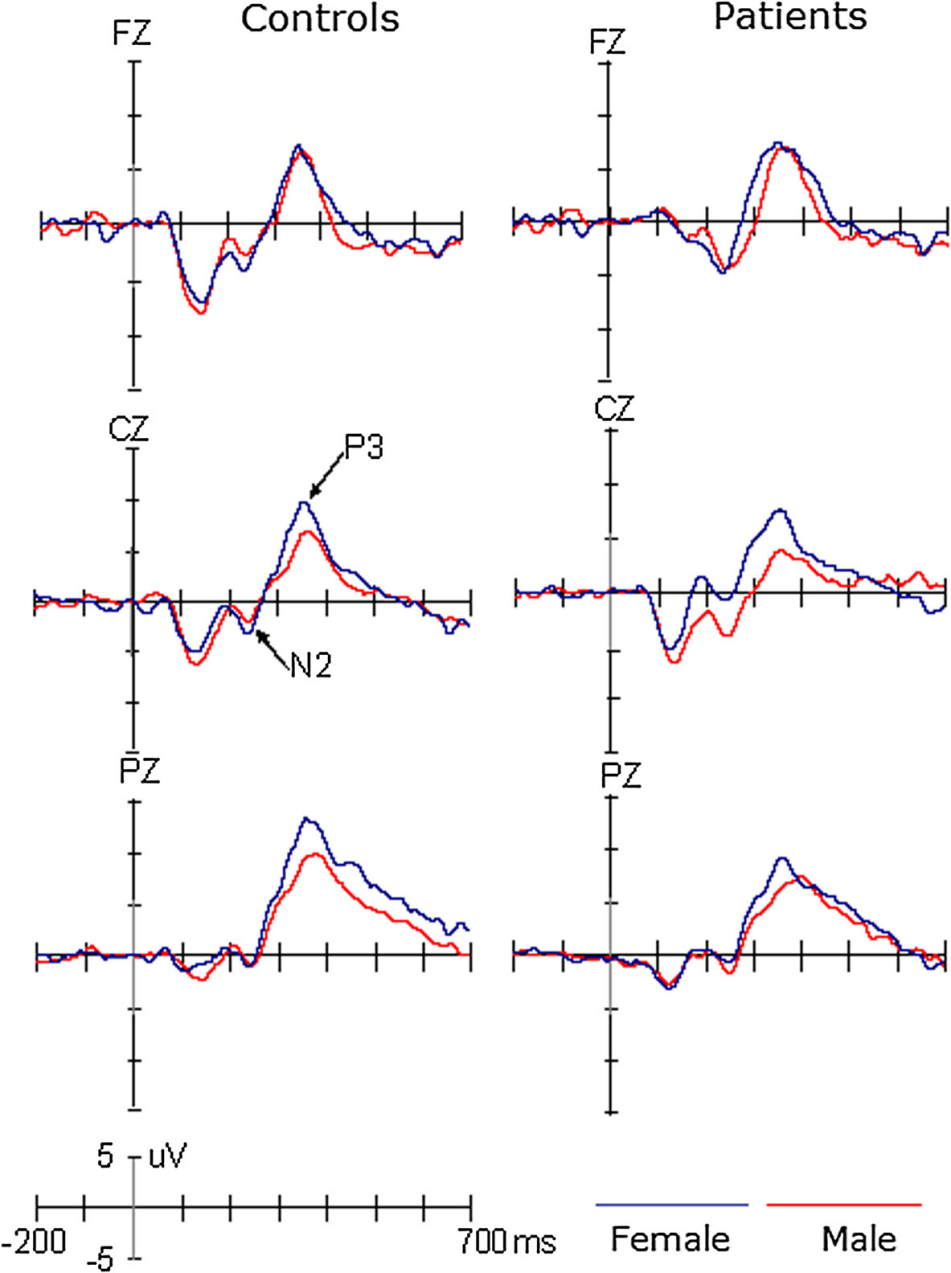
The grand averaged ERPs elicited by target stimuli in patients and controls, respectively.

**Figure 2:**
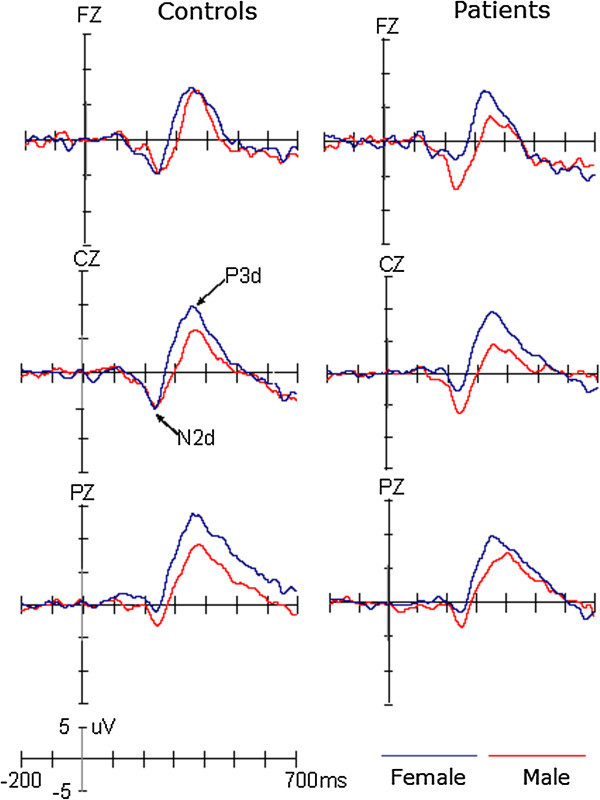
The difference waveforms by subtracting ERPs in response to standard stimuli from ERPs in response to target stimuli in patients and controls, respectively.

The peak amplitudes and latencies of two ERP components, N2 and P3, were measured relative to the pre-stimulus baseline period (see Figure 1). The negative peak between 200 and 300 ms and the positive peak between 300 and 500 ms were used to define the N2 and P3 components, respectively. To reliably observe the target effect, the difference waveform was obtained by subtracting ERPs in response to standard stimuli from ERPs in response to target stimuli (see Figure 2). The mean amplitudes were measured between 200 and 300 ms for the N2d (i.e., the N2 target effect) and 300 and 500 ms for the P3d (i.e., the P3 target effect), respectively.

### Data analysis

The measurements of N2 and P3 components were analyzed using repeated-measures analysis of variance (ANOVA), with Stimulus (target, standard) and Site (Fz, Cz, and Pz for P3; Fz and Cz for N2) as within-subject factors and with Gender (female, male) and Group (migraine, control) as between-subject factors.

For N2d and P3d components, the ANOVA was conducted with Site (Fz, Cz, and Pz for P3d; Fz and Cz for N2d) as within-subject factors and with Gender (female, male) and Group (migraine, control) as between-subject factors. The degrees of freedom were corrected using the Greenhouse–Geisser epsilon.

## Results

### Behavioral data

For the accuracy, neither the group effect (control: 99%; patients: 99.25%) nor the gender effect (female: 99.25%; male: 99.11%) was significant (Fs < 1). The gender × group interaction was not significant (F(1,53) < 1).

The response speed was not affected by the group (323 ms and 333 ms for controls and patients, respectively; F(1,53) < 1), nor by the gender (331 ms and 345 ms for males and females, respectively; F(1,53) < 1). The gender × group interaction was not significant (F(1,53) < 1).

### ERP data

#### N2 and N2d components

The amplitudes of N2 component showed significant main effect of Site (F(1,53) =10.184, p < 0.005, η2 = 0.16), indicating larger N2 at Fz (-3.90 μV) than Cz (-2.16 μV) site. Although neither the group (F(1,53) < 1) nor the gender (F(1,53) = 2.34, p > 0.05) effect was significant, there was a significant interaction of Gender × Group (F(1,53) = 5.785, p < 0.025, η2 = 0.398). Post-hoc tests revealed that, while there was no significant difference between female patients and female controls (p = 0.313), the mean amplitude of N2 was larger for male patients (-6.06 μV) than male controls (-1.94 μV; p < 0.025, η2 = 0.129). In the control group the gender effect was not significant (p = 0.532), but for patients the N2 was larger for males than for females (p < 0.01). No other effects reached significant level (ps > 0.1).

The analysis of N2d amplitudes did not demonstrate the significant main effect of Site (F(1,53) < 1) or Group (F(1,53) < 1). The gender effect was marginally significant, F(1,53) = 4.0, p = 0.051, but qualified by the two-way interaction of Group × Gender, F(1,53) = 4.467, p < 0.05, η2 = 0.14. Post-hoc tests revealed that the N2d was larger in male patients (-7.40 μV) than in male controls (-4.37 μV, p < 0.05) and that, although the gender effect was not significant in normal controls (p = 0.907), the N2d amplitude was larger for male patients than for female patients (-2.22 μV; p < 0.01). We did not observe any significant effects or interactions in the N2 or N2d latencies (ps > 0.1).

#### P3 and P3d components

Across conditions, both P3 and P3d showed significant main effects of Site (F(2,106) = 16.52, p < 0.001, η2 = 0.388 for P3 and F(2,106) = 11.44, p < 0.001, η2 = 0.178 for P3d), indicating a centro-parietal scalp distribution with a maximize of 8.77 μV for P3 and 8.81 μV for P3d at Pz. The amplitudes of P3 and P3d were smaller for patients (P3, 4.43 μV; P3d, 6.25 μV) than for controls (P3, 7.64 μV; P3d, 8.21 μV), F(1,53) = 5.68, p < 0.025, η2 = 0.397 for P3 and F(1,53) = 4.22, p < 0.05, η2 = 0.12 for P3d, respectively. Although the amplitudes of P3 did not appear to be significantly affected by Gender (F(1,53) = 2.92, p = 0.093), across groups, the P3d were larger for female than for male participants (F(1,53) = 5.73, p < 0.025, η2 = 0.10). No other effects reached significant level (ps > 0.1). The latencies of the P3 and P3d components did not show any significant effects (ps > 0.1).

## Discussion

In this study, we used a traditional auditory oddball paradigm, in which participants were required to press a button for the infrequent target stimulus while ignoring the frequent non-target standard stimulus while ignoring the frequent non-target standard stimulus, and focused on P3 component, which is a generic name for a variety of relatively late positive components with a centro-parietal or centro-frontal midline distribution [17,18]. In addition, we will also investigate the N2, a frontal-central distributed negativity that reflects the stimulus evaluation response including action monitoring, the early target-selection and response preparation [19]. If there were gender effects on cognitive function in patients, it should be reflected by a modulation of the N2 and/or P3 components.

The present study found the P3 component was larger for female than for male participants, for both the patient and control groups. Compared with the control group, there was a decrease in the amplitude of P3 for patients, and this reduction was not modulated by gender. Although there was no gender effect on the amplitudes of N2 in the control group, they were larger for male than for female migraine patients and larger for healthy male participants.

This study replicated the results of previous studies by finding that females had larger P3 amplitudes than males, regardless of their migraine history. Importantly, the P3 amplitudes in migraine patients were significantly decreased in comparison with the control group, as was the case in previous studies [17]. It has been widely accepted that P3 is a neural signature of attention and/or the amount of working memory required for appropriately responding to environmental stimuli [20]. Using a similar paradigm to the present study, Wang et al. found smaller P3 for migraine patients than for control subjects. Interestingly, recent studies revealed that the reduction of the P3 amplitude was also evident when using the passive oddball paradigm, in which the infrequent novel stimuli were examined [13]. This indicates that migraine patients exhibit a deficit in the frontal function involved in automatic attention switching [14]. Overall, the present pattern of P3 components provided new evidence for the dysfunction of cognitive function in migraine patients.

Although we did not find an influence of gender on the P3 component, the N2 component was larger for male than for female patients (note that there were no gender effects in control participants) and was larger for male patients than for male controls. Generally, the target-related N2 component indicates action monitoring, early target-selection and response preparation [21-25]. Therefore, it is possible that male patients have a super-sensitive cerebral function that is relevant to early target-selection and response preparation. There have been neuroimaging studies providing some relevant evidences for the hypersensitivity of cortical function in patients with migraine. For example, Martín H, et al. studied light sensitivity and photophobia in migraineurs by assessing the response to light stimuli with fMRI-BOLD of the occipital cortex and found that migraineurs during interictal periods showed hyperxcitability of the visual cortex with a wider photoresponsive area [26]. Similarly, it was found that migraine patients showed significantly higher blood oxygen level-dependent siginal intensities in the brain areas including limbic structures and the rostral pons in response to olfactory stimulation during spontaneous and untreated attacks [27]. Recently, Woolf & Salter developed a conceptual framework for the contribution of plasticity in primary sensory and dorsal horn neurons to the pathogenesis of pain. They identified distinct forms of plasticity that elicit pain hypersensitivity by increasing gain [28,29]. However, it should be noted that the above studies did not investigate the gender effects of the hypersensitivity of the cortical function. In other word, to our knowledge there was no evidence for larger neutral plasticity of the brain in male than female migraineurs. Especially, the present findings showed that the N2 component, indeed, did not differ between female patients and control participants. Therefore, it is necessary in the future to determine whether the super neutral plasticity is specific to male participants, at least at the N2 level.

Interestingly, one recent neuroimaging study showed dysfunctional organization in the resting functional network of the brain that was more evident in female migraine patients [4]. In addition, the migraine may cause abnormal brain structure and brain function, which depends on the patients’ gender. For example, compared with male migraineurs, female patients had thicker posterior insula and precuneus cortices [3]. To date, converging evidence shows that the incidence of migraine in females is about three times as high as in males, and that estrogen could be the main cause of this gender difference [30]. However, we did not observe gender differences in the P3 components and consequently, the differences of estrogen between male and female cannot account for the different cognitive function across patients’ genders.

## Conclusion

Before concluding, we would like to reiterate the procedural decisions that constrained the interpretation of the present findings. First, we did not measure ERPs in conjunction with other neuropsychological investigations. Second, the small cohort limited the examination of the effect of the age and the differences between migraine with and without aura. Nevertheless, we took steps to ensure that the sample was as homogeneous as possible by choosing only young participants, which undoubtedly reduced the impact of confounders on our analysis. Third, we did not check for the occurrence of an attack after the recording session. Finally, although the present study found group effects on the N2 and P3 components, the data were unable to reveal the spatial distribution of these abnormalities because of the limitation on recorded electrode sites (Fz, Cz, and Pz). Therefore, in order to reveal the gender differences of brain topographical distributions it is necessary to record multi-electrode sites with possible source analysis in the future.

These limitations notwithstanding, our findings emphasize the importance of considering gender when studying cognitive processing in migraine patients, and provide further empirical support that a gender effect exists.

## Competing interests

All authors declare there are non-financial competing interests (political, personal, religious, ideological, academic, intellectual, commercial or any other) in relation to this manuscript.

## Authors’ contributions

M.D. RW, ZD, XC, MZ, FY, XZ and WJ carried out the studies. And RW drafted the manuscript. M.D. RW participated in the design of the study and performed the statistical analysis. Professor SY, the PI of this study, conceived of the study and participated in its design and helped to draft the manuscript. All authors read and approved the final manuscript.
